# Identification of Radiation-Induced miRNA Biomarkers Using the CGL1 Cell Model System

**DOI:** 10.3390/bioengineering9050214

**Published:** 2022-05-16

**Authors:** Jayden Peterson, Christopher D. McTiernan, Christopher Thome, Neelam Khaper, Simon J. Lees, Douglas R. Boreham, Tze Chun Tai, Sujeenthar Tharmalingam

**Affiliations:** 1 School of Natural Sciences, Laurentian University, Sudbury, ON P3E 2C6, Canada; jpeterson1@laurentian.ca (J.P.); cthome@nosm.ca (C.T.); dboreham@nosm.ca (D.R.B.); tc.tai@nosm.ca (T.C.T.); 2 Medical Sciences Division, NOSM University, 935 Ramsey Lake Rd., Sudbury, ON P3E 2C6, Canada; chrisdmctiernan@gmail.com; 3 Biomolecular Sciences Program, Laurentian University, Sudbury, ON P3E 2C6, Canada; 4 Health Sciences North Research Institute, Sudbury, ON P3E 2H2, Canada; 5 Medical Sciences Division, NOSM University, 955 Oliver Rd., Thunder Bay, ON P7B 5E1, Canada; nkhaper@nosm.ca (N.K.); slees@nosm.ca (S.J.L.); 6 Department of Biology, Lakehead University, Thunder Bay, ON P7B 5E1, Canada

**Keywords:** miRNA, epigenetics, low-dose radiation, next-generation sequencing, miRNAome, CGL1, biomarkers

## Abstract

MicroRNAs (miRNAs) have emerged as a potential class of biomolecules for diagnostic biomarker applications. miRNAs are small non-coding RNA molecules, produced and released by cells in response to various stimuli, that demonstrate remarkable stability in a wide range of biological fluids, in extreme pH fluctuations, and after multiple freeze–thaw cycles. Given these advantages, identification of miRNA-based biomarkers for radiation exposures can contribute to the development of reliable biological dosimetry methods, especially for low-dose radiation (LDR) exposures. In this study, an miRNAome next-generation sequencing (NGS) approach was utilized to identify novel radiation-induced miRNA gene changes within the CGL1 human cell line. Here, irradiations of 10, 100, and 1000 mGy were performed and the samples were collected 1, 6, and 24 h post-irradiation. Corroboration of the miRNAome results with RT-qPCR verification confirmed the identification of numerous radiation-induced miRNA expression changes at all doses assessed. Further evaluation of select radiation-induced miRNAs, including miR-1228-3p and miR-758-5p, as well as their downstream mRNA targets, *Ube2d2*, *Ppp2r2d*, and *Id2*, demonstrated significantly dysregulated reciprocal expression patterns. Further evaluation is needed to determine whether the candidate miRNA biomarkers identified in this study can serve as suitable targets for radiation biodosimetry applications.

## 1. Introduction

Biodosimetry is the measurement of biological response to radiation. The gold standard in the field of biological dosimetry is the dicentric chromosome assay (DCA), which measures the incidence of dicentric chromosomes [1]. DCA has a predicting error of ±0.5 Gy which is adequate for high-dose radiation (HDR) exposures [2]. However, DCA is unreliable in the low-dose radiation (LDR) range (below 100 mGy) [2]. The cytokinesis-block micronucleus (CBMN) assay is also used for biodosimetry applications. The CBMN assay assesses the formation of micronuclei in binucleated cells derived from peripheral lymphocytes [3]. These micronuclei are formed when chromosomal structures are damaged, forming chromosome fragments that segregate separately during cell division. Unfortunately, both DCA and CBMN are low-throughput methodologies and are ineffective for estimating LDR exposures [4]. This emphasizes the need for new biodosimetry techniques that are reliable, sensitive, high-throughput, and minimally invasive.

microRNAs (miRNAs) are becoming more prevalent within the biomarker field as these biomolecules can be accurately analyzed to predict prognosis while being easily accessible due the fact of its abundance within patient serum [5]. miRNAs also demonstrate remarkable stability in a wide range of biological fluids [6]. In addition, miRNAs are resistant to multiple freeze–thaw cycles and extreme pH fluctuations; therefore, samples can be readily transported [7]. As such, these traits make miRNA key molecules when looking for specific biological markers for radiation exposures.

miRNAs are small non-protein coding RNA molecules approximately 20 nucleotides in length that act to post-transcriptionally modulate the gene expression profile of cells [8]. Two miRNAs with different cellular targets result per miRNA gene and are denoted with either 3p or 5p to indicate their origin. One of these two mature miRNA strands is then bound to Argonaute (AGO), forming a ribonucleoprotein complex known as the RNA-induced silencing complex (RISC) [9]^.^ This complex uses the miRNA to bind to the complementary mRNA target and the AGO protein cleaves the mRNA strand. Despite having two viable miRNAs per gene, 3p and 5p, one of the species is usually more frequently integrated into the RISC complex than the other [10]. This more frequently integrated species is dependent on the particular gene itself and does not relate directly to the 3p or 5p end [11]. Taken together, complementary binding of miRNA to target mRNA prevents its translation and, therefore, reduces downstream protein expression.

Numerous studies have demonstrated that HDR exposure significantly alters miRNA profiles, resulting in downstream changes to various radiation-related molecular pathways including DNA damage repair, apoptosis, and cell cycle arrest [12,13,14]. Overall, the vast majority of the research on radiation and miRNA involves studies examining radiation therapy effects on cancer treatment and are thus HDR focused [15,16,17]. Another limitation of these studies is the lack of next-generation sequencing (NGS)-based miRNAome technology within the context of radiation-induced miRNA profiling [18,19,20]. While many articles investigated the interaction of one or more miRNAs, this selective approach can potentially introduce bias. NGS-based miRNAome profiling would ensure a more comprehensive investigation of the biological system and potentially lead to previously missed novel miRNA interactions resulting from the more focused approach. Taken together, further research is needed to comprehensively profile dose-dependent radiation-induced miRNA that can serve as candidate biomarkers for biodosimetry applications.

The primary aim of this study was to identify novel radiation-induced miRNA biomarkers at various dose ranges spanning LDR and HDR exposures. In this study, the entire miRNAome of the CGL1 cells were analyzed post-radiation exposure to identify dose-dependent radiation-induced miRNA expression changes. The CGL1 cell line is a preneoplastic nontumorigenic model resulting from the hybridization of a normal male skin GM0077 fibroblast cell with the malignant HeLa cervical cancer cell [21,22]. The CGL1 cells are nontumorigenic and demonstrate a normal fibroblast-like phenotype and transcriptome profile [23]. The CGL1 cell model system has been used extensively to investigate the effects of radiation-induced cellular transformation and tumorigenicity [24].

## 2. Materials and Methods

### 2.1. Cell Culture

The CGL1 cell line was grown in a humid environment at 37 °C with 5% CO_2_ [21]. The CGL1 cells were cultured using 1X Minimal Essential media (Corning, Manassas, VA, USA) with the addition of 50 U penicillin–streptomycin (GIBCO, Grand Island, NY, USA) and 5% calf serum (Hyclone, Logan, UT, USA).

### 2.2. Cell Irradiation

The CGL1 cells were exposed to X-rays in triplicate at 10, 100, or 1000 mGy and collected at 1, 6, and 24 h post-irradiation. Irradiations were performed on an X-RAD 320 irradiation cabinet (Precision X-ray, Madison, CT, USA) operated at 320 kV and 12.5 mA with a 2 mm Al filter. Briefly, cells were grown in a T25 flask (Life Technologies, Carlsbad, CA, USA) and plated to ensure 80% confluence at the desired endpoint. These sample sets consisted of three different timepoints post-irradiation (i.e., 1, 6, and 24 h) to assess the time-dependent expression of the various radiation-induced miRNA. These timepoints allowed for the analysis of acute (1 h), intermediate (6 h), and prolonged effects (24 h). At each of these timepoints, the cells were split into 4 different irradiation groups: the sham irradiation control and 10, 100, and 1000 mGy. This range of dose was selected to evaluate LDR effects (10 and 100 mGy) and a higher dose of radiation (1000 mGy), which is well known to induce DNA damage effects. These samples were irradiated on ice with cold PBS to ensure that no-dose rate effects were observed. The cells were irradiated using three different programs to ensure similar exposure times among the different doses. In short, the 10 mGy dose was irradiated at a dose rate of 5.6 mGy/min, the 100 mGy dose was irradiated at a dose rate of 138 mGy/min, and the 1000 mGy dose was irradiated at a dose rate of 1500 mGy/min. Following irradiation, the samples were re-incubated in media until the desired timepoint, at which point the cells were lysed and collected using 1 mL TRIzol per T-25 flask.

### 2.3. Total RNA Extraction

Total RNA was extracted using the TRIzol extraction method according to the manufacturer’s instructions. In brief, 0.2 mL of chloroform was added to 1 mL TRIZOL samples and vortexed prior to being centrifuged at 12,000× *g* for 20 min. The top aqueous layer containing the RNA was transferred into another tube, while the remaining DNA and protein layer were stored for future use. The RNA was precipitated out of the aqueous layer with the addition of 0.3 mL of isopropanol and centrifuged at 12,000× *g* for 15 min. Afterwards, the supernatant was decanted, and the remaining RNA pellet was washed with 1 mL of 70% ethanol and centrifuged at 7500× *g* for 5 min. Finally, the supernatant was removed, and the resulting RNA pellet was resuspended in 30 µL of DEPC water. The resulting total RNA samples were assessed to ensure both an adequate concentration and purity using a Nanodrop (Nanodrop One, Thermo Fisher Scientific, Madison, WI, USA), assuring the 260/280 nm absorbance ratio of the samples was greater than 1.8.

### 2.4. cDNA Synthesis

From the total RNA samples, one of two reverse transcription reactions were performed depending on whether mRNA or miRNA was to be analyzed. In brief, total RNA was first purified of DNA contaminants using a DNAse kit (MilliporeSigma, Oakville, ON, CA) before proceeding to reverse transcription reactions. The key difference between mRNA and miRNA cDNA preparation was that for the analysis of miRNAs, the samples were first subjected to a poly-A tail extension and annealed to an oligo-dT primer with a known sequence at the 3′ end (5′-GCATAGACCTGAATGGCGGTAAGGGTGTGGTAGGCGAGACATTTTTTTTTTTTTTTTTTTT-3′). This additional step before the cDNA synthesis serves to elongate the miRNA and produce a suitable nucleotide length for RT-qPCR analysis, without which RT-qPCR would have been otherwise impossible, as the miRNA are too similar in length to design RT-qPCR primers. For preparation of cDNA from mRNA, random hexamers were used. The resulting products were reverse transcribed using the Promega M-MLV Reverse Transcriptase kit (Madison, WI, USA).

### 2.5. miRNAome Profiling via Next-Generation Sequencing

An NGS analysis was conducted on the irradiated samples; however, based on various studies in the literature, 6 h post-stimulation was found to be an optimal timepoint, where most miRNA are expected to be expressed and, therefore, of most importance [25]. As such, the 6 h timepoint RNA samples were chosen for miRNAome analysis. The remaining two timepoints, 1 and 24 h, were analyzed subsequently using RT-qPCR and compared to the 6 h results. This allowed for the determination of the time-course for the expression of the dysregulated miRNA. For the sample preparation, the irradiated total RNA samples were treated with the QIAseq miRNA Library kit (QIAGEN Sciences, Germantown, MD, USA), which serves to select smaller RNA within a sample and prepare these RNA sequences for miRNAome profiling. In brief, following the collection of total RNA using the method described earlier, two ligation reactions were accomplished to add adapter sequences to the 5′ and 3′ end of the miRNA. A reverse transcription reaction integrating the UMI adaptors into the resulting cDNA was completed. Finally, a barcode index was added to the end of each of the sample conditions. Samples were then pooled together and sent out for sequencing at the Donnelly Sequencing Centre at the University of Toronto. The samples were sequenced on the NovoSeq6000 platform (Illumina) and consisted of a 75-base length read for a total of 10 million reads per sample. Previous reports have shown that 5 million sequencing reads per sample is adequate for total human miRNAome profiling [26].

### 2.6. RT-qPCR Primer Design and Validation

Despite the NGS methodology being an excellent technique capable of whole miRNAome profiling, false positives can occur based on statistical probability as well as other contributing factors such as library amplification and sequencing bias [27]. As such, a common practice to verify the integrity of the obtained sequencing results is to utilize a secondary confirmatory technique on randomly selected miRNA targets. Here, randomly selected miRNAs were cross-verified using RT-qPCR analysis.

RT-qPCR primers were designed *in-house* using the primer blast software offered by the National Center for Biotechnology Information (NCBI). For the design of the mRNA primers, the full-length mRNA transcript sequences were used [28]. However, for the miRNA primers, a modified input sequence was used consisting of the miRNA sequence appended to the oligodT adapter sequence. 

The potential primers were screened for the combination of melting temperature, GC content, and lack of self-complementarity to obtain the optimal primer pairs for each gene. These primers were verified across a range of temperatures between 54 and 64 °C and run at various concentrations to ensure their amplification was optimal. The primers for the mRNA gene expression analysis were considered valid if their amplification reaction efficiency was uniform, had an R^2^ value of >0.99, and an amplification efficiency of 90–110%. For the miRNA, the amplification efficiency was relaxed to 70–130% in order to provide more flexibility than usual due to the limitations when designing miRNA primers; these miRNA primers usually correspond to the miRNA sequence itself with minimal design flexibility. The miRNA cDNA products were qPCR amplified using a universal reverse primer for the known end sequence (5′-GCATAGACCTGAATGGCGGTA-3) common to all of the modified miRNA and a forward primer specific to the miRNA in question. Validated primer information can be found in the appendix (Table A1 for miRNA and Table A2 for mRNA).

### 2.7. RT-qPCR

The RT-qPCR procedure was performed in 15 µL reaction volume ensuring a final volume of 1X qPCR master mix (LUNA, New England Biolabs, Ipswich, MA, USA) and 600 nM for both primers. The protocol was repeated for 40 cycles: 95 °C for 15 s and 60 °C for 30 s, after which the fluorescence was measured. A melt curve was performed at the end of the 40-cycle run to ensure that the resulting amplicon was unique. Once complete and the raw fluorescence data were obtained, the cycle threshold (Cq) data were analyzed using the QuantStudio^TM^ Design and Analysis Software v1.5.1 (Applied Biosystems). Samples were normalized to the geometric mean of two control housekeeping genes: RPS18/GAPDH for mRNA and SNORD48/U6 for miRNA. The relative expression of the genes was calculated utilizing the ΔΔCT method with the following formula: 2^ΔΔCT^ = 2^(ΔCT gene − ΔCT housekeeping genes)^. The average 2^ΔΔCT^ and standard error of the means (SEMs) were calculated [29].

### 2.8. Statistical Analysis

For the NGS data, the irradiated samples were first normalized with their sham control using the DEseq2 methodology package previously described [30]. Genes that had a *p*-value less than 0.05, a gene count greater than 50, and a fold change of at least 1.5 were considered significant.

For the RT-qPCR-based experiments, the different doses within a timepoint were compared to one another by performing a one-way ANOVA followed by Tukey’s post hoc analysis using Jamovi (*p*-values < 0.05 were considered significant).

## 3. Results

### 3.1. Identification of Dysregulated miRNA 6 h Post-Irradiation

The miRNAome analysis of CGL1 cells exposed to 10, 100, and 1000 mGy doses 6 h post-radiation is presented in Table 1. The miRNAome analysis identified a total of 2256 miRNAs within the CGL1 cells. Of these, 38 miRNAs were significantly dysregulated compared to the sham controls, demonstrating radiation-induced expression profiles.

### 3.2. Validation of the miRNAome Results Via RT-qPCR Analysis

**Figure 1:**
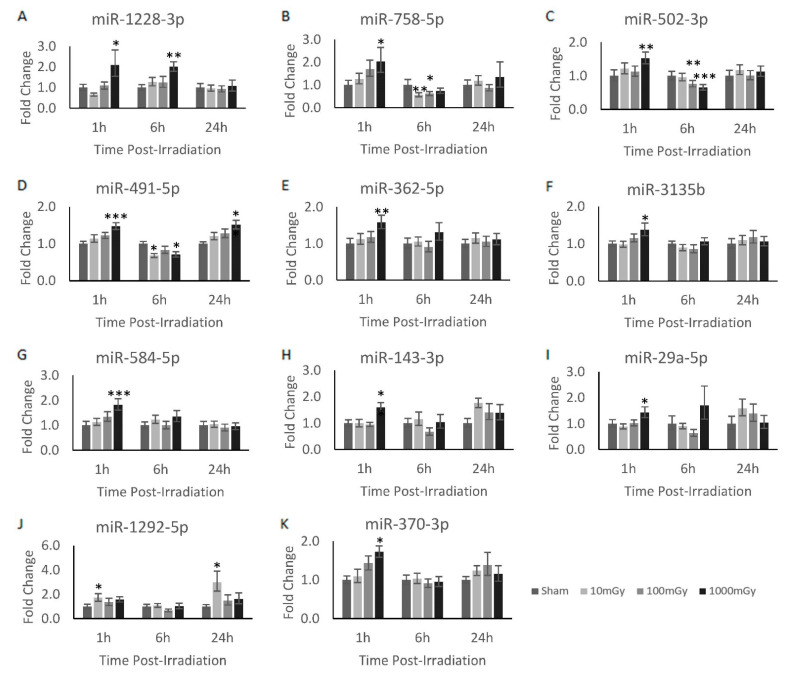
Radiation-induced miRNA dysregulation in CGL1 cells. Various doses and timepoints were analyzed via RT-qPCR to obtain the temporal expression profile of the analyzed miRNAs in CGL1 cells. Cells were irradiated at 10, 100, and 1000 mGy and collected 1, 6, and 24 h post-irradiation. All irradiation doses were normalized to their own timepoint’s sham condition to obtain the relative fold change. All miRNA shown had at least one dose and timepoint that was considered significant; however, most of the significant findings were shown to be at the 1000 mGy dose. Significance is denoted by an asterisk, where * *p* < 0.05, ** *p* < 0.01, and *** *p* < 0.001.

To verify the validity of the significantly dysregulated miRNA identified from the miRNAome results, a secondary quantitative analysis was performed on the irradiated sample sets. Here, the validation of the miRNAome results were corroborated with RT-qPCR analysis. In addition, the 1 and 24 h timepoints were analyzed along with the 6 h timepoint to identify temporal expression patterns. As such, the adapted RT-qPCR technique for the quantification of miRNA described earlier was performed on the samples. The RT-qPCR primers were designed for all of the radiation-induced miRNAs identified in Table 1. However, only 19 of the total primers passed validation due to the limitations in designing primers for the short miRNA sequence (Table A1). From these validated primers, 11 miRNAs were found to be significantly dysregulated as identified via miRNAome and RT-qPCR methodologies across the different experimental conditions: miR-1228-3p, miR-758-5p, miR-502-3p, miR-491-5p, miR-362-5p, miR-3135b, miR-584-5p, miR-143-3p, miR-29a-5p, miR-1292-5p, and miR-370-3p (Figure 1). Of these miRNAs, miR-362-5p, miR-3135b, miR-584-5p, miR-143-3p, miR-29a-5p, and miR-370-3p shared the same temporal expression profile and were significantly upregulated at the 1000 mGy dose at the 1 h timepoint (Figure 1E–K). In fact, all 11 miRNAs, except for miR-1292-5p, were significantly upregulated at 1000 mGy 1 h post-irradiation. Moreover, miR-1292-5p demonstrated dysregulation uniquely at the 10 mGy dose, where it can be shown to be significantly increased at both the 1 and 24 h timepoints (Figure 1J). Another interesting trend was shown with miR-1228-3p, which demonstrated consistent upregulation at 1000 mGy between the 1 and 6 h timepoints (Figure 1A). The remaining miRNAs, miR-758-5p, miR-502-3p, miR-491-5p, shared a similar expression profile, wherein they showed a decrease in expression during the 6 h timepoint at 1000 mGy relative to the sham. Moreover, these miRNAs also demonstrated significant dose-dependent decreases compared to the sham: miR-758-5p showed a decrease at the 10 and 100 mGy doses (Figure 1B), miR-502-3p decreased at the 100 and 1000 mGy doses (Figure 1C), and miR-491-5p was reduced at the 10 and 1000 mGy doses (Figure 1D). Additionally, miR-491-5p also showed a significant increase at the 1000 mGy dose at the 24 h timepoint.

### 3.3. mRNA Gene Targets of miR-1228-3p and miR-758-5p Showed Reciprocal Expression

**Figure 2:**
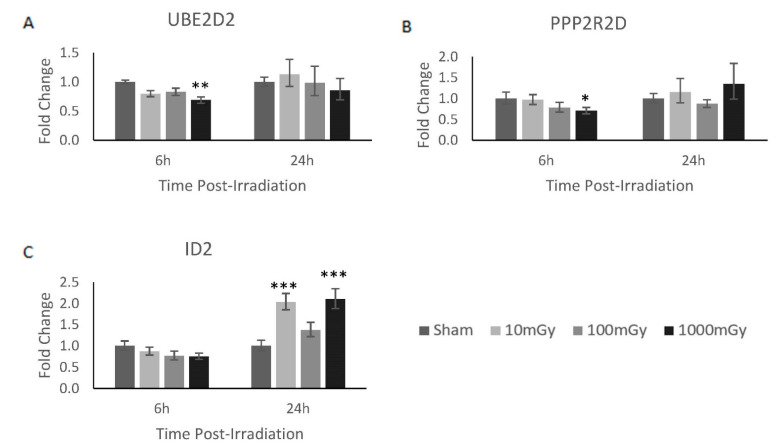
mRNA gene targets of miR-1228-3p and miR-758-5p showed reciprocal expression. Of the validated possible mRNA interaction shown in Table 2, three mRNA gene targets demonstrated a reciprocal gene expression pattern to their corresponding miRNA. In brief, (**A**) *Ube2d2* and (**B**) *Ppp2r2d* demonstrated a reciprocal expression pattern to miR-1228-3p at 6 h, whereas (**C**) *Id2* showed a reciprocal expression pattern to miR-758-5p at 24 h. Significance is denoted with asterisks, where * = *p* < 0.05, ** = *p* < 0.01, and *** = *p* < 0.001.

To better understand the underlying mechanism of action for these various significantly dysregulated miRNAs, their various downstream gene targets were identified based on the predicted interaction using the miRBD database and previously documented in the literature (Table 2) [31]. By examining all these mRNA targets, it can be determined if the change in miRNA expression demonstrates reciprocal effects on the expression of the mRNA targets. Here, reciprocal miRNA and mRNA expression demonstrated biologically relevant interactions [32]. However, for this analysis, it is important to note that miRNAs are typically induced much faster and last much longer than their mRNA counterparts; as such, the various timepoint expressions across mRNA and miRNA may not be a direct temporal relationship [33]. From the analysis of these mRNA targets, three significant miRNA/mRNA reciprocal interactions were identified. Here, miR-1228-3p was upregulated, whereas its mRNA targets, *Ube2d2* and *Ppp2r2d*, were found to be significantly downregulated 6 h post-1000 mGy irradiation (Figure 2A,B). In addition, miR-758-5p was downregulated, while its mRNA target *Id2* was found to be significantly upregulated at 24 h post-10/1000 mGy irradiation (Figure 2C).

## 4. Discussion

The overall miRNAome results showed 38 dysregulated genes across various radiation doses (Table 1). As predicted, an increasing number of genes were shown to be dysregulated when exposed to increasing levels of radiation [34]. To compare radiation dose-dependent expression patterns, 2 miRNAs were dysregulated at 10 mGy, 8 miRNAs were dysregulated at 100 mGy, and 29 miRNAs were dysregulated at 1000 mGy. Of those, 11 miRNAs were validated based on RT-qPCR analysis, and a further 2 miRNAs were shown to have reciprocal response to their predicted mRNA targets (Figure 2).

As mentioned, three mRNA/miRNA target sets were validated from the above results in terms of reciprocal expression patterns. In short, the expression of genes *Ube2d2* and *Ppp2r2d* were inversely related to the expression of miR-1228-3p, whereas *Id2* gene expression was inversely related to its target of miR-758-5p. To further explore the role of these radiation-induced mRNA changes within the CGL1 cells, their mechanism of action is discussed below based on the known literature.

**Figure 3:**
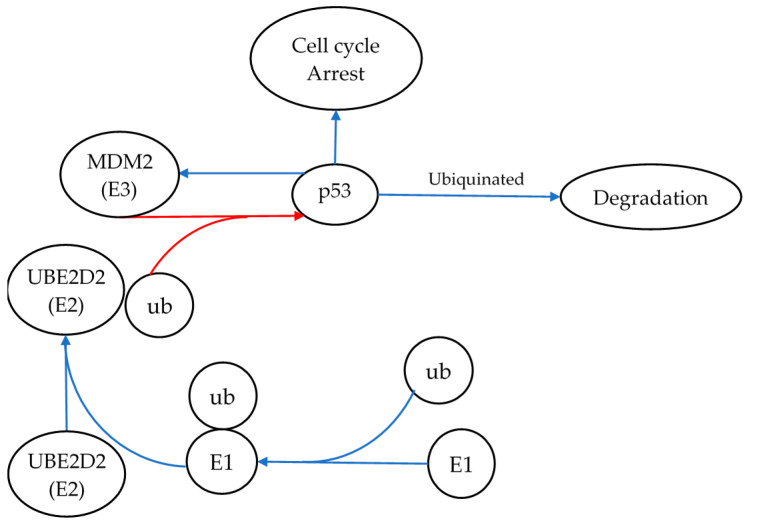
Ube2d2’s role in p53 degradation, and the signaling pathway demonstrating the role of miR-1228-3p and its target Ube2d2 in regulating the degradation of p53. Here, Ube2d2 is shown transferring ubiquitin (ub) onto p53 via MDM2 and, thus, targeting p53 for degradation. miR-1228-3p inhibits the translation of *Ube2d2*. Therefore, radiation-induced expression of miR-1228-3p should reduce Ube2d2-mediated degradation of p53. Thus, increased expression of miR-1228-3p is expected to result in elevated p53 levels. The blue arrows represent pathway activation, whereas the red arrows illustrate pathway inhibition. E1 represents ubiquitin-activating enzymes that catalyze the first step in the ubiquitination reaction.

Ube2d2 is a component of the protein ubiquitination pathway, a protein modification often associated with protein degradation [35]. The main function of Ube2d2 is to accept ubiquitin from the E1 complex and to catalyze the reaction between ubiquitin and other proteins [35]. Of special interest within our study was its role in the degradation of p53 via its interaction with MDM2 as illustrated in Figure 3 [35]. When paired with the knowledge that p53 is constitutively expressed and degraded via this system, it can be concluded that the levels of p53 within the cell may change in response to the expression of *Ube2d2* [31,36]. In short, p53 levels should decrease when Ube2d2 is highly expressed and vice versa. Given that miR-1228-3p inhibits the translation of *Ube2d2*, radiation-induced expression of miR-1228-3p should therefore reduce Ube2d2-mediated degradation of p53. Therefore, increased expression of miR-1228-3p is expected to result in elevated p53 levels. In this specific case, where miR1228-3p was upregulated following 1 Gy irradiation, it can be inferred that the overall level of active p53 protein is likely elevated via a reduction in its degradation. Therefore, miR-1228-3p is potentially a radiation-induced master regulator of p53 activity and further investigation would be needed to conclude if p53 protein levels are increased in response to radiation-mediated upregulation of miR-1228-3p. Following that assumption, p53-related activities, including cell cycle arrest, DNA damage repair, and apoptosis, would likely be enhanced as a result of increased miR-1228-3p levels [37]. 

The second validated target of miR-1228-3p is a subunit of the protein phosphatase 2A (PP2A) complex, which is a ubiquitously expressed phosphatase that is generally associated with tumour suppressive activity [38]. The complex is a tri-heteromeric protein in which subunits A and C are responsible for its structural and enzymatic activity, whereas the many types of B subunits are responsible for its specificity and cellular localization [39]. PPP2R2D is a specific B subunit variant (B55 delta) of PP2A that serves to regulate its function and is associated with a key role within the cell cycle by controlling the exit of mitosis via its inhibitory effect on CDK1 [36]. In short, it is highly expressed when the cell is in interphase and found in lower quantities during mitosis [40]. However, a recent finding has also shown Ppp2r2d to have a potential oncogenic role within the cell, as it has repeatedly been shown to be upregulated in gastrointestinal cancer cells and its inhibition is deleterious to the cells [41]. This study demonstrated that the Ppp2r2d subunit was correlated with higher levels of p-mTOR protein, thus potentially affecting the cell proliferative state; however, a mechanism for this correlation could not be identified [41]. This discovery aligns with previous knowledge demonstrating that while PP2A acts primarily as a tumour suppressor, certain subunits, including Ppp2r2d, show the ability to positively regulate signalling pathways such as the MAPK signalling cascade [42]. These findings indicate that a reduction in Ppp2r2d via miR-1228-3p may lead to a decrease in proliferative signals after exposure to 1 Gy of radiation. This coincides with the expected radiation-induced stress response discussed with Ube2d2, where the cell initially undergoes cell cycle arrest post-radiation to allow progression of various repair pathways.

**Figure 4:**
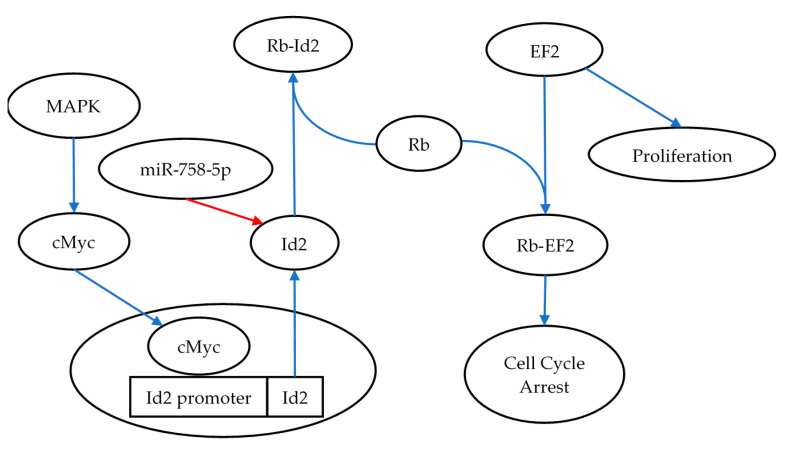
Id2’s role in reversing cell cycle arrest, illustrating the role of miR-758-5p in regulating Id2 and the consequence of upregulating Id2 via the activation of the MAPK signaling pathway. As part of the recovery process following radiation exposure, Id2 is expressed and binds to Rb. This interaction prevents binding to its other binding partner EF2, thus inducing cell proliferation [46]. The blue arrows in the figure represent pathway activation, whereas the red arrows illustrate pathway inhibition.

In addition to miR-1228-3p, this study identified reciprocal expression of miR-758-5p and *Id2* (Inhibitor of DNA Binding 2). ID2 is a transcriptional regulator that is capable of binding to other transcription factors and preventing their binding to DNA, thus negatively regulating their activity [43]. As such, it can affect a diverse set of signalling pathways, which may be of interest in the context of radiation biology including proliferation, cell cycle arrest, and apoptosis pathways [44]. One such study showed results consistent with those presented here, where Id2 demonstrated an increase in expression following 24 h post-irradiation [45]. This study established that Id2 had the ability to reverse the cell cycle arrest induced by gamma irradiation, demonstrated a protective role when cells were exposed to irradiation, and promoted cell proliferation [45]. Taken together these finding may indicate that subsequent decreases in miR-758-5p at later timepoints post-irradiation trigger an increase in ID2, which promotes the return of the cell to normal functions following the irradiation-induced cell cycle arrest (Figure 4).

Taken together, miR-1228-3p and miR-758-5p appear to be the most promising radiation-induced miRNAs identified from this study. However, all of the radiation-induced miRNAs presented in Table 1 from the miRNAome analysis are potential targets to pursue further as possible biomarkers for LDR exposures. Other promising results include miR-4443, which was shown to be dysregulated at 10 and 100 mGy doses and may potentially show high sensitivity to radiation. In fact, all miRNAs found within the 10 and 100 mGy LDR range are novel radiation-induced miRNAs and should be further assessed for biodosimetry applications of LDR exposures.

The results from this study demonstrate that a single miRNA may not be sufficient to serve as a biomarker for a broad range of doses that spans LDR and HDR exposures. This study points to the use of dose-range specific miRNAs that can be assessed together to develop biodosimetry biomarkers to accurately identify a broad range of radiation dose exposures. In this scenario, a multiplex RT-qPCR design encompassing various dose-range specific primers for radiation-induced miRNAs may serve as a reliable approach for biodosimetry applications.

In addition, as shown in Figure 1, most of the miRNAs were dysregulated at the 1 h timepoint, whereas minimal lasting miRNA dysregulation was identified at the 24 h timepoint. This would potentially be a problem when determining the applicability of miRNA-based biodosimeter biomarkers, as the temporal window for detection should be as large as possible. Therefore, miRNAs, such as miR-1292-5p and miR-491-5p, that demonstrated prolonged dysregulation at 24 h post-irradiation may be of note. However, it is possible that the radiation-induced miRNAs may have been secreted as exosomes, contributing to the lack of elevated expression at 24 h post-irradiation with the cell.

Additionally, this study indicates that mRNA targets may also serve as potential radiation biomarkers. Here, we identified reduced expression of miR-758-5p and an upregulation of its mRNA target *Id2* 24 h post-radiation. Interestingly, Id2 expression has been previously identified in the literature as a radiation-induced biomarker [43].

This study provides a technical framework for identifying radiation-induced miRNA biomarkers using NGS-based miRNAome technology. Similar studies need to be performed in various cell types and in vivo models to elucidate reliable radiation-induced miRNA biomarkers. In addition, it is important to verify whether the radiation-induced miRNAs are also detectable in the extracellular environment in exosome fractions [47]. Given that serum and urine samples are likely the ideal choice for sampling radiation exposures, identification of released miRNA is crucial for developing reliable miRNA biomarkers for biodosimetry application.

## 5. Conclusions

In conclusion, this study identified numerous potential candidate radiation-induced miRNAs at various dose ranges. The results of the miRNAome study identified 38 radiation-induced miRNA that were dysregulated within the irradiated samples. From there, a total of 11 miRNA were further verified via RT-qPCR-based miRNA expression analysis. Here, two miRNAs demonstrated reciprocal gene expression with its predicted mRNA targets: miRNAs 1228-3p and 758-5p and their corresponding mRNA targets *Ube2d2*/*Ppp2r2d* and *Id2*, respectively. The most promising was the interaction of the miR-1228-3p target *Ube2d2* and its role in p53 degradation. This finding suggests that radiation-induced expression of miR-1228-3p may promote higher levels of p53. Elevated p53 suggests a stress response typically expected after radiation exposures.

Altogether, the results discussed in this paper revealed novel potential miRNA biomarkers within the CGL1 cell line using miRNAome sequencing. Although this study requires additional investigation to validate its findings, the data presented here represents a good foundation for future investigations.

## Figures and Tables

**Table 1:** Dysregulated miRNA in CGL1 cells 6 h post-irradiation.

Dose (mGy)	miRNA	Fold Change	*p*-Value
10	miR-3120-3p	11.3	0.042
	miR-4443	1.9	0.048
100	miR-4443	2.3	0.009
	miR-362-5p	2.1	0.034
	miR-148b-5p	2.0	0.043
	miR-423-3p	1.9	0.016
	miR-500b-5p	1.7	0.040
	miR-502-3p	1.6	0.040
	miR-125b-1-3p	1.6	0.041
	miR-495-5p	-1.7	0.040
1000	miR-3168	8.8	0.0008
	miR-671-5p	5.3	0.003
	miR-6835-5p	4.2	0.001
	miR-5694	4.1	0.009
	miR-491-5p	3.9	0.005
	miR-2054	3.6	0.003
	miR-4668-5p	3.5	0.002
	miR-6069	3.4	0.019
	miR-23a-5p	3.3	0.003
	miR-3135b	2.8	0.015
	miR-22-3p	2.7	0.022
	miR-29a-5p	2.7	0.045
	miR-665	2.5	0.007
	miR-296-3p	2.1	0.0004
	miR-6813-3p	2.1	0.008
	miR-1292-5p	2.0	0.011
	miR-4271	1.9	0.006
	miR-1228-3p	1.8	0.043
	miR-193a-5p	1.7	0.007
	miR-370-3p	1.7	0.020
	miR-758-5p	1.7	0.044
	miR-584-5p	-2.1	0.021
	miR-598-3p	-2.2	0.050
	miR-449c-3p	-2.4	0.042
	miR-181a-2-3p	-2.5	0.022
	miR-10b-5p	-;2.7	0.035
	miR-143-3p	-2.8	0.034
	miR-889-3p	-2.8	0.041
	miR-100-5p	-2.9	0.028

A list of miRNAs from the miRNAome study that were found to be significant across all doses at the 6 h timepoint after three cut-offs were applied to the initial data: *p*-value < 0.05, fold change >1.5, and sequencing read count >50 reads in all three biological replicates.

**Table 2:** A list of top mRNA targets for selected radiation-induced miRNA.

miRNA	mRNA Targets
miR-362-5p	*Rbm27*, *Trim50*, *Sgip1*, *Plagl2*, *Prkacb*, *Luc7l3*, *Edem1*, *Cyp1b1*, *Rmi1*, *Mrpl35*, *Pik3c2b*, *Cyld*, *Gas7*
miR-491-5p	*Sema6a*, *Chrnb2*, *Pogk*, *Ksr2*, *Samd4b*, *Optc*, *Cpne5*, *Foxp4*, *Msn*, *Igf2*, *Smad3*, *Bcl2l1*, *Egfr*, *Notch3*, *Mmp9*, *Igf2bp1*, *Wnt3a*, *Capns1*
miR-495-5p	*Cttn*, *Ncoa2*, *Znf281*, *Tnpo1*, *Hnrnpd*, *Sbno1*, *Dipk2a*, *Mcub*, *Nol4l*, *Camta1*, *Cdk6*, *Mta3*
miR-502-3p	*Adamts3*, *Kctd9*, *Fbn2*, *Sec63*, *Zfx*, *Dapk1*, *Smim13*, *Napil5*, *Csde1*, *Set*, *Olfm4*
miR-584-5p	*Usp6nl*, *Avpr1a*, *Hsd11b1*, *Galnt15*, *Gbp5*, *Cd200*, *Ppm1a*, *Nphp1*, *Rock1*, *Mmp14*, *Kcne2*, *Wwp1*, *Cdk16*
miR-758-5p	*Slc20a2*, *Id2*, *Nufip2*, *Ptp4a1*, *Tox4*, *Setd5*, *Phactr1*, *Rtkn*, *Dsg3*, *Csnk1a1l*, *Cd36*, *Zbtb20*, *Cbx5*
miR-1228-3p	*Ppp2r2d*, *Ube2d2*, *Irx2*, *Znf554*, *Nfia*, *Socs6*, *Rabgef1*, *Tjp1*, *Tor1aip1*, *Zbtb44*, *Moap1*, *Csnk2a1*, *Plac8*
miR-3135b	*Lrrc27*, *Fmnl3*, *Ttc21b*, *Castor3*, *Xpo7*, *Ppm1a*, *Dnm1l*, *Kdm3b*, *Rbp1*, *Faap20*, *Pten*, *Golph3*

A list of potential miRNA gene targets was obtained from the miRBD database for each of the miRNA genes in Figure 1. The miRBD database presented a score denoting the likelihood of the miRNA interacting with various mRNA. From that scoring, the top dozen ranked mRNA gene targets for each miRNA were chosen for further analyses.

## Data Availability

The data that support the findings of this study are available from the corresponding author upon a reasonable request.

## Appendix A

**Table A1:** RT-qPCR primers for miRNA gene expression profiling.

**miRNA**	**miRNA forward Primer**	**Tm**
Oligo-dT adapter	GCATAGACCTGAATGGCGGTAAGGGTGTGGTAGGCGAGACATTTTTTTTTTTTTTTTTTTT	65
universal reverse	GCATAGACCTGAATGGCGGTA	60
SNORD48	TGATGACCCCAGGTAACTCTGA	60
miR-16-5p	ATTGAAACCTCTAAGAGTGGA	60
U6	GTG CTC GCT TCG GCA GCA CAT AT	60
miR-423-3p	AGCTCGGTCTGAGGCCC	60
miR-191-5p	CAACGGAATCCCAAAAGCAGC	60
miR-6134	UGA GGU GGU AGG AUG UAG A	60
miR-6082	GAATACGTCTGGTTGATCCAAAA	57
miR-208a-5p	AGCTTTTGGCCCGGGTTATAC	61
miR-7112-3p	CATCACAGCCTTTGGCCCTA	60
miR-6762-3p	UGGCUGCUUCCCUUGGUCUCCAG	60
miR-6882-3p	CTCTCCTCTTGCCTGCAGAA	60
miR-200a-5p	TCTTACCGGACAGTGCTGGA	61
miR-604	GCTGCGGAATTCAGGACAAAA	60
miR-2355-5p	TCCCCAGATACAATGGACAAAA	57
miR-4692	UCAGGCAGUGUGGGUAUCAGAU	60
miR-378e	ACTGGACTTGGAGTCAGGAAA	59
miR-4535	GGACCTGGCTGGGACAAAA	60
miR-1915-3p	CGACGCGGCGGGAAA	60
miR-1291	CCTGACTGAAGACCAGCAGTAAA	60
miR-19a-5p	AGTTTTGCATAGTTGCACTACAAA	58
miR-5008-3p	TCCCAGGGCCTCGCAAA	61
miR-4417	TGGGCTTCCCGGAGGG	60
miR-1537-3p	AAAACCGUCUAGUUACAGUUGU	60
miR-3168	GAGUUCUACAGUCAGAC	60
miR-5186	AGAGAUUGGUAGAAAUCAGGU	60
miR-4680-5p	AGAACTCTTGCAGTCTTAGATGT	57
miR-554	AGTCCTGACTCAGCCAGTAAAAA	60
miR-6845-3p	CCUCUCCUCCCUGUGCCCCAG	60
miR-6125	GCGGAGCGGCGGAAAA	61
miR-3616-3p	CGAGGGCATTTCATGATGCAG	60
miR-615-3p	UCCGAGCCUGGGUCUCCCUCUU	60
miR-3152-3p	UGUGUUAGAAUAGGGGCAAUAA	60
miR-4693-3p	UGAGAGUGGAAUUCACAGUAUUU	60
miR-4320	GGGAUUCUGUAGCUUCCU	60
miR-6783-5p	GGAAAAGTCCTGATCCGGAAAAA	59
miR-33b-5p	GUGCAUUGCUGUUGCAUUGC	60
miR-4662a-3p	AAAGAUAGACAAUUGGCUAAAU	60
miR-8082	GATGGAGCTGGGAATACTCTGAA	60
miR-4758-5p	GCCGGTGGGGCTGAAAA	60
miR-8071	GGACTGGAGTGGGTGGAAAAA	60
miR-4477b	AUUAAGGACAUUUGUGAUUGAU	60
miR-34a-5p	TGGCAGTGTCTTAGCTGGTTG	60
miR-182-5p	TTTGGCAATGGTAGAACTCACACT	60
miR-525-3p	AAGGCGCTTCCCTTTAGAGC	60
hsa-miR-492	AGGACCTGCGGGACAAGA	60
hsa-miR-4535	GTGGACCTGGCTGGGAC	60
miR-877-5p	GTAGAGGAGATGGCGCAGG	60
miR-133b	TTTGGTCCCCTTCAACCAGC	60
miR-205-3p	GATTTCAGTGGAGTGAAGTTC	60
miR-1	ACATACTTCTTTATATGCCCAT	60
miR-19b	AGTTTTGCAGGTTTGCATCCAG	60
miR-93-5p	AAGTGCTGTTCGTGCAGGTAG	60
miR-132-5p	ACCGTGGCTTTCGATTGTTAC	59
miR-671-5p	CTGGAGGGGCTGGAGAAAAA	60
miR-628-3p	TCTAGTAAGAGTGGCAGTCGAA	58
miR-125b-1-3p	ACGGGTTAGGCTCTTGGGA	60
miR-6797-5p	GAAGGGGCTGAGAACAGGAAA	60
miR-6739-5p	TGGGAAAGAGAAAGAACAAGTAAAA	57
miR-6823-3p	GCCTCTCCTTCCCTCCAGAAA	61
miR-449c-3p	GCTAGTTGCACTCCTCTCTGT	59
miR-328-3p	CCTCTCTGCCCTTCCGTAAA	59
miR-103a-3p	CAGCATTGTACAGGGCTATGAA	58
miR-4721	CTCCAGGTGACGGTGGAAAAA	60
miR-589-3p	CAGAACAAATGCCGGTTCCC	60
miR-769-3p	GGATCTCCGGGGTCTTGGT	61
miR-27a-3p	CACAGTGGCTAAGTTCCGCA	61
let-7g-5p	TGAGGTAGTAGTTTGTACAGTT	60
let-7a-5p	TGAGGTAGTAGGTTGTATAGTT	60
miR-296-5p	AGGGCCCCCCCTCAATCCTGT	60
miR-4443	TTGGAGGCGTGGGTTTT	60
miR-3120-3p	ACAGCAAGTGTAGACAGGCAA	60
miR-423-3p	AGCTCGGTCTGAGGCCCCTCAGT	60
miR-362-5p	CCTTGGAACCTAGGTGTGAGT	60
miR-148b-5p	AGTTCTGTTATACACTCAGGCAA	60
miR-495-5p	GAAGTTGCCCATGTTATTTTCG	60
miR-6835-5p	AGGGGGUAGAAAGUGGCUGAAG	60
miR-4668-5p	AGGGAAAAAAAAAAGGAUUUGUC	60
miR-491-5p	GTGGGGAACCCTTCCATGAG	60
miR-4271	GGGGGAAGAAAAGGTGGGG	60
miR-665	GGAGGCTGAGGCCCCTAAA	60
miR-193a-5p	TCTTTGCGGGCGAGATGAAA	60
miR-22-3p	AGCTGCCAGTTGAAGAACTGTA	60
miR-1228-3p	CACACCTGCCTCGCCC	60
miR-181a-2-3p	CACTGACCGTTGACTGTACCA	60
miR-100-5p	ACCCGTAGATCCGAACTTGTG	60
miR-143-3p	TGAGATGAAGCACTGTAGCTC	60
miR-10b-5p	ACCCTGTAGAACCGAATTTGTGA	60

A total list of miRNA primers tested during the course of the secondary validation step of the obtained NGS results. The sequences and their optimal melting temperature can be found above.

**Table A2:** RT-qPCR primers for mRNA gene expression profiling.

**mRNA Primer**	**Sequence (5′→3′)**	**Length**	**Product (bp)**	**Tm**
PPP2R2D_F_Hu_1	GTCAAGGACAGGGCAGACTTC	21	136	60
PPP2R2D_R_Hu_2	AGCTGTTCTCAGCTGTTCTATCA	23		
UBE2D2_F_Hu_1	CGTTTTGCCCGATCCACAAG	20	145	60
UBE2D2_R_Hu_1	GTCCCGTGCCAGATCATTCA	20		
IRX2_F_Hu_1	CACCAAGATGACCCTCACCC	20	119	60
IRX2_R_Hu_1	CGTCCTCGTCCTCATCTTCG	20		
ZNF554_F_Hu_1	CTGCAGTACTGTGCTGATCCA	21	137	60
ZNF554_R_Hu_1	ACGTCTCTGTCCAGCCTTTG	20		
NFIA_F_Hu_1	TGCCGAATCGATTGCAACTTC	21	83	60
NFIA_R_Hu_1	GGCTGGTTCTCAGATTCGCT	20		
SOCS6_F_Hu_1	GGCCGCCTCCGGAAAAT	17	81	60
SOCS6_R_Hu_1	ACATCTGGAGAGGCTGCAAG	20		
RABGEF1_F_Hu_1	GCGGTGACCTGGACCAC	17	82	60
RABGEF1_R_Hu_1	TTCCCAAAGTGATCTCGCCC	20		
TJP1_F_Hu_1	TGGTCTGTTTGCCCACTGTT	20	150	60
TJP1_R_Hu_1	TCTGTACATGCTGGCCAAGG	20		
TOR1AIP1_F_Hu_1	AAGTCCTCTAGTGCAACGCC	20	82	60
TOR1AIP1_R_Hu_1	ATCTTGGCTTGAGGCACTCC	20		
ZBTB44_F_Hu_1	ACGAGTGCAAAACATGTGGC	20	72	60
ZBTB44_R_Hu_1	GGTTCAGACTCCTCAGGTGC	20		
Moap1_F_Hu_1	AGGCCCTTCTCCAGGCAATA	20	70	60
Moap1_R_Hu_1	TGCCATATCCCTTCGTGGTT	20		
Csnk2a1_F_Hu_1	ATCGCCGCCATATTGTCTGT	20	109	60
Csnk2a1_R_Hu_1	CAGCTGGGGGTAAGACCTTG	20		
PLAC8_F_Hu_1	CAGAAGGAGAGCCATGCGTA	20	76	60
PLAC8_R_Hu_1	AACCCACATGTTCTGAGAGGC	21		
SLC20A2_F_Hu_1	TCTCGGCCTAATGTGGTAGGA	21	138	60
SLC20A2_R_Hu_1	CTCCCGATCTGGGAAAGCTG	20		
ID2_F_Hu_1	ATCCTGTCCTTGCAGGCTTC	20	81	60
ID2_R_Hu_1	ACCGCTTATTCAGCCACACA	20		
NUFIP2_F_Hu_1	ATGTCCATTTTGCTTGCCTGG	21	149	60
NUFIP2_R_Hu_1	CCCAATTCAGGTGGGGTCTG	20		
PTP4A1_F_Hu_1	CTGTGAGCTCTTAAGACTTGCTT	23	73	58
PTP4A1_R_Hu_1	CACTGCTGCTGGGAATTATGA	21		
TOX4_F_Hu_1	CTGACGATCACAGGGCCTTC	20	142	60
TOX4_R_Hu_1	GGCCAACCACATCTGAGACA	20		
SETD5_F_Hu_1	CTGTGACAAGTGCAGGGGAA	20	95	60
SETD5_R_Hu_1	CTGTTGCACTGCTATCCCCA	20		
PHACTR1_F_Hu_1	TGTTCATTTGTGCTTGCGGG	20	74	60
PHACTR1_R_Hu_1	CCCTTTCAACAGGACACGGT	20		
RTKN_F_Hu_1	CGAGTGAAGTGTGACTCCGT	20	90	60
RTKN_R_Hu_1	TTCCAGACAGGAAACCAGCC	20		
DSG3_F_Hu_1	GACTCCTTCGGAAAGCAGCA	20	138	60
DSG3_R_Hu_1	GGGGAAGAGCCCCATCATTG	20		
CSNK1A1L_F_Hu_1	CCCTGGGGTTTGCAAATTGT	20	138	60
CSNK1A1L_R_Hu_1	TCTTCACAGGTAAGCAGGCG	20		
CD36_F_Hu_1	CCACACACTGGGATCTGACA	20	132	60
CD36_R_Hu_1	TCTGCAGGAAAGTCCTACACTG	22		
ZBTB20_F_Hu_1	TCCTGACAAATGCTAGAACGGA	22	86	60
ZBTB20_R_Hu_1	CCACCCGGCTGAGTAATCTC	20		
CBX5_F_Hu_1	GGGAGGCCCCTCCTGTTAG	19	72	60
CBX5_R_Hu_1	AAGACTAAGGCCACCAGGTC	20		
CTTN_F_Hu_1	GACAAATGTGCCCTTGGCTG	20	111	60
CTTN_R_Hu_1	CTGCCTCTCCGACTGAACAC	20		
NCOA2_F_Hu_1	CCCTCCCTCTACCACAGTCA	20	87	60
NCOA2_R_Hu_1	CAGAGTCCTCTGAGAAGGCG	20		
ZNF281_F_Hu_1	ATGACCACCATGGCACTGAG	20	70	60
ZNF281_R_Hu_1	TCTGGCTTTGGCCTTTTTGC	20		
TNPO1_F_Hu_1	TGCCCGGCCGTTTGAAG	17	121	60
TNPO1_R_Hu_1	GCTCGTCAGGTTTCCACTCA	20		
HNRNPD_F_Hu_1	GCCATTCAAACTCCTCCCCA	20	89	60
HNRNPD_R_Hu_1	GTCCCAGCTAAGGCCTCCTA	20		
SBNO1_F_Hu_1	CAATGCCTACCCCGTCAGTT	20	72	60
SBNO1_R_Hu_1	CTGCTTCGGTCTCCAAACCT	20		
DIPK2A_F_Hu_1	GTGGGTGTGAGACATCCTAGC	21	74	60
DIPK2A_R_Hu_1	CACGACAAGTGGGGTCTGC	19		
MCUB_F_Hu_1	GGAGGATGCTCCAGAGGGG	19	119	60
MCUB_R_Hu_1	CTTCACACGCAAAACCTGGG	20		
NOL4L_F_Hu_1	GCCAAAACCAAGACGGTGAC	20	124	6
NOL4L_R_Hu_1	CCCAGAACTGGAACTTGCCT	20		
CAMTA1_F_Hu_1	GAAAACAAGCCGGAAGAGCG	20	81	60
CAMTA1_R_Hu_1	ATAGGTGGCACGGTGTTGAG	202		
cdk6_F_Hu_1	CTGCAGGGAAAGAAAAGTGCAA	22	95	60
cdk6_R_Hu_1	CTCCTCGAAGCGAAGTCCTC	20		
MTA3_F_Hu_1	GTCCTCCCCCTCCGCTC	17	131	60
MTA3_R_Hu_1	CTGGGGACTGCCCAATTCAT	20		
TRIM50_F_Hu_1	GGCATCTAACTGGAGCGACA	20	85	60
TRIM50_R_Hu_1	CCAAGCCATCCACACTCACT	20		
SGIP1_F_Hu_1	TGGAATTCCTTCAGGCGGAC	20	73	60
SGIP1_R_Hu_1	ACGATTCCAGGTCCCAGCTA	20		
PLAGL2_F_Hu_1	ACAATGCACCGCACAATGG	19	138	60
PLAGL2_R_Hu_1	CCTCCAACGCAGCTTTCAGA	20		
PRKACB_F_Hu_1	GCTAGCAGTAAGAGCTGGTGT	21	75	60
PRKACB_R_Hu_1	TGAACCTGGCAAGGAGCAAA	20		
LUC7L3_F_Hu_1	GGTCAATGGGACCAGTGAAGA	21	147	60
LUC7L3_R_Hu_1	CGCTGCACTGTCAAACAGTAA	21		
EDEM1_F_Hu_1	ACAACTACATGGCTCACGCC	20	91	60
EDEM1_R_Hu_1	AGATTTGAAGGGTCCCCGC	19		
CYP1B1_F_Hu_1	GCTGTGAGGAAACCTCGACT	20	121	60
CYP1B1_R_Hu_1	GAGTCTCTTGGCGTCGTCAG	20		
RMI1_F_Hu_1	GCGGTTCCTGTCCTTACAGT	19	86	60
RMI1_R_Hu_1	ACTGCTCAGAAATGGCCCTG	20		
MRPL35_F_Hu_1	TGCAAAGAAATTGGGTCTGTGT	22	141	60
MRPL35_R_Hu_1	TGAAGGGCCACCCTTAAACC	20		
PIK3C2B_F_Hu_1	CCACCATAGAGATGGCGTCC	20	134	60
PIK3C2B_R_Hu_1	TGGGCGCCTGATTCTTCTAC	20		
Cyld_F_Hu_1	CCCCCTTTCTAGGGTGAGGA	20	98	60
Cyld_R_Hu_1	TTCAGCAACGTGGTGTCCAT	20		
GAS7_F_Hu_1	AGCCAACGAGTCTCTGCTTC	20	79	60
GAS7_R_Hu_1	GCCGTCTCTGGGGTGC	16		
LRRC27_F_Hu_1	CTCGCCAGCGCTTCAGT	17	102	60
LRRC27_R_Hu_1	TAGGAGCTGCTTCCCTCCAT	20		
FMNL3_F_Hu_1	GAGTCGGGACTCGGGGAG	18	130	60
FMNL3_R_Hu_1	CTCTCCAGGTTGCCCATCG	9		
TTC21B_F_Hu_1	TGCGTCTTCCTTTAGGCTGC	20	87	60
TTC21B_R_Hu_1	TCTTCAATTCCTGCGAGTCCA	21		
CASTOR3_F_Hu_1	AGCTTTTCCAGACCAGGCAT	20	76	60
CASTOR3_R_Hu_1	CTAGGGGCTGATGTGCCAAA	20		
XPO7_F_Hu_1	GCAATCACAGACGTCACAAGG	21	126	60
XPO7_R_Hu_1	TGCTTCAATGAGGAAGGCTGT	21		
PPM1A_F_Hu_1	CTGCTCCGGACCTAGAGGAT	20	124	60
PPM1A_R_Hu_1	CAGCCTTGCATGCTGCTTAG	20		
DNM1L_F_Hu_1	TCACCCGGAGACCTCTCATT	20	91	60
DNM1L_R_Hu_1	TCTGCTTCCACCCCATTTTCT	21		
KDM3B_F_Hu_1	CTGCGCACTCGAGCCTG	17	114	60
KDM3B_R_Hu_1	CCAGGAGTGTTGCTTCCAGT	20		
RBP1_F_Hu_1	CCGCTACAATGGATCCTCCC	20	96	60
RBP1_R_Hu_1	GGAAATGAGCGCCCTCCG	18		
FAAP20_F_Hu_1	GGGTCCCCTTCTCCACTGTA	20	78	60
FAAP20_R_Hu_1	CTGGCAGGAGCTGGAGATG	20		
PTEN_F_Hu_1	CTGCAGAAAGACTTGAAGGCG	21	70	58
PTEN_R_Hu_1	TGCTTTGAATCCAAAAACCTTACT	24		
GOLPH3_F_Hu_1	TGTTTCCTCATGACTGCCCC	20	80	60
GOLPH3_R_Hu_1	CGATCCGGGTTTCCGTGTTA	20		
ADAMTS3_F_Hu_1	CAAGCATTCTCCGCGCTAAC	20	147	60
ADAMTS3_R_Hu_1	GGAGCGAGAAGGTGCTGTAA	20		
KCTD9_F_Hu_1	CCCAAGAACGGAAAGGTGGT	20	88	60
KCTD9_R_Hu_1	TGGTGGCTTTTATGCCGAGT	20		
FBN2_F_Hu_1	GTTTTCTGCCAGTCATCCAGC	21	142	60
FBN2_R_Hu_1	AGCTGCTTTGGCTTCGATCT	20		
SEC63_F_Hu_1	GGACATAAATAGGGCAATCCACT	23	119	58
SEC63_R_Hu_1	CCCTCTCACTCCTGGGTTTT	20		
ZFX_F_Hu_1	ACCCTAGTGGAGTGTTGGCT	20	123	60
ZFX_R_Hu_1	TGAACCACTGAAGGGAGTCG	20		
DAPK1_F_Hu_1	TTCGGAGTGTGAGGAGGACA	20	149	60
DAPK1_R_Hu_1	GGGAACACAGCTAGGGAGTG	20		
SMIM13_F_Hu_1	AGTGGGTGAAAATTCCCGCT	20	94	60
SMIM13_R_Hu_1	CCCTGGTAAACACTCAGCCC	20		
NAP1L5_F_Hu_1	CTCCTAGACCTCTGCGGCTT	20	147	62
NAP1L5_R_Hu_1	GCTGTCACAGTCTCCACCCT	20		
CSDE1_F_Hu_1	CGCTGAGCTGTTGGGTATGA	20	78	60
CSDE1_R_Hu_1	ACGAGGTTTGTTCCTTGCCT	20		
set_F_Hu_1	AGTCTCAGTGTTCAGCCTGC	20	78	60
set_R_Hu_1	GGCCATGCTGTTAGGGAAGT	20		
OLFM4_F_Hu_1	AAATGCTCGAGAGTTGCGGA	20	133	60
OLFM4_R_Hu_1	CACAGCAATCGTGTTGGTGG	20		
USP6NL_F_Hu_1	TGGAAGGGAAACAATGGGGC	20	144	60
USP6NL_R_Hu_1	CATGTCCTCAGTACGGTCCC	20		
AVPR1A_F_Hu_1	TGGGCGCCTTTCTTCATCAT	20	75	60
AVPR1A_R_Hu_1	AGGGTTTTCCGATTCGGTCC	20		
HSD11B1_F_Hu_1	TGCCTGCTTAGGAGGTTGTAG	21	90	60
HSD11B1_R_Hu_1	AAAAGCCATCCGACAGGGAG	20		
GALNT15_F_Hu_1	ACCCAGATGGCTTATTGCCT	20	126	60
GALNT15_R_Hu_1	TACCAGCCAGGGACTGAGTT	20		
GBP5_F_Hu_1	AGTTCTGCTTGACACCGAGG	20	149	60
GBP5_R_Hu_1	GCAGTAGGTCGATAGCACCC	20		
CD200_F_Hu_1	TCCAGGAGCAAGGATGGAGA	20	76	60
CD200_R_Hu_1	GACCCAAACCAGGCTGTAGG	20		
PPM1A_F_Hu_1	CTGCTCCGGACCTAGAGGAT	20	124	60
PPM1A_R_Hu_1	CAGCCTTGCATGCTGCTTAG	20		
NPHP1_F_Hu_1	CCCACTTCTCCACTCCACAC	20	76	60
NPHP1_R_Hu_1	AACTTTCCACCGTGCAGTCT	20		
Rock_F_Hu_1	CCCTTTGCTTTCGCCTTTCC	20	150	60
Rock1_R_Hu_1	GAGGTGCTTCAGTCTAGCGG	20		
mmp14_F_Hu_1	GGGTCTTCGTTGCTCAGTCA	20	145	60
mmp14_R_Hu_1	AACATTCGAGAGGCACAGGG	20		
kcne2_F_Hu_1	ACGGGAACACTCCAATGACC	20	105	60
kcne2_R_Hu_1	TGGATGGTGGCCTTCGATTC	20		
WWP1_F_Hu_1	TGCTACTTTTAGCAAACTGGGC	22	128	58
WWP1_R_Hu_1	TTAAGAAGTCAGTTCCATGGCT	22		
cdk16_F_Hu_1	TTGGGCCGTTGGCTGTTC	18	70	60
cdk16_R_Hu_1	GGCTCGCGGCACAGAG	16		
SEMA6A_F_Hu_1	CTTACAACACAGTGTATGGGCA	22	81	60
SEMA6A_R_Hu_1	CATACCCCTCTTGAGCCGTC	20		
CHRNB2_F_Hu_1	GAAAGTTCGGCTCCCTTCCA	20	115	60
CHRNB2_R_Hu_1	GCCATCATAGGAGACCACGG	20		
POGK_F_Hu_1	TGGGAAGTTTTGACGGAGCA	20	113	60
POGK_R_Hu_1	CGGGTGATCATGTCTGGCTT	20		
KSR2_F_Hu_1	AAAGCACTCCAAACCGTGGA	20	133	60
KSR2_R_Hu_1	ACTCTTTACACACCGGCTCC	20		
SAMD4B_F_Hu_1	CCTTCCTACTGGGCAGATGAG	21	101	60
SAMD4B_R_Hu_1	CCAGAAGTGGACATGGGGTA	20		
OPTC_F_Hu_1	TGTCTTCAACCTGGCCTGTC	20	70	60
OPTC_R_Hu_1	AGTACACAAGCCCATCCAGG	20		
CPNE5_F_Hu_1	TGTGTCCAACGGTGGTGTC	19	113	60
CPNE5_R_Hu_1	CCAGCTTGTTGGCACAGAAC	20		
FOXP4_F_Hu_1	AGCTGATTTGCTGCAGGGAT	20	100	60
FOXP4_R_Hu_1	GAAGGACACCTGGGAATGGG	20		
MSN_F_Hu_1	GCCCAAAACGATCAGTGTGC	20	88	60
MSN_R_Hu_1	AAATAGCTGCTTCCCGGTGG	20		
IGF2_F_Hu_1	TCGCCGAACCAAAGTGGATTA	21	148	60
IGF2_R_Hu_1	GTGGGAGAGACAGAGTGAACG	21		
Smad3_F_Hu_1	CCGGGGGTTGGACTTTCCT	19	70	60
Smad3_R_Hu_1	CAGAAGTTTGGGTTTCCGCA	20		
Bcl2l1_F_Hu_1	AGGCGGATTTGAATCTCTTTCTCT	24	129	60
Bcl2l1_R_Hu_1	GGGCTCAACCAGTCCATTGT	20		
Egfr_F_Hu_1	GACAGGCCACCTCGTCG	17	106	60
Egfr_R_Hu_1	CCGGCTCTCCCGATCAATAC	20		
Notch3_F_Hu_1	TCTAGGTAAGGTGGGGAGTGG	21	70	60
Notch3_R_Hu_1	TGGGAGCTCAAGTTAGCCCT	20		
Mmp9 _F_Hu_1	GTACTCGACCTGTACCAGCG	20	92	60
Mmp9 _R_Hu_1	AGAAGCCCCACTTCTTGTCG	20		
Igf2bp1_F_Hu_1	AGCTCCTTTATGCAGGCTCC	20	111	60
lgf2bp1_R_Hu_1	CCGGGAGAGCTGTTTGATGT	20		
Wnt3a_F_Hu_1	CTCCTCCCTGGAGCTAGTGT	20	138	60
Wnt3a_R_Hu_1	AATCTGTAGCCCCGCCTCTG	20		
capns1_F_Hu_1	CGGACGCTGCGGGAG	15	72	60
capns1_R_Hu_1	TCACTGCGCCGCACAC	16		

A total list of mRNA primers tested, consisting of both predicted mRNA targets for the studied miRNA and the genes chosen for use in the DNA damage panel. The sequences of both primer pairs and their optimal melting temperature can be found above.

## References

[1] Rothkamm K., Beinke C., Romm H., Badie C., Balagurunathan Y., Barnard S., Bernard N., Boulay-Greene H., Brengues M., de Amicis A. (2013). Comparison of established and emerging biodosimetry assays. Radiat. Res..

[2] Lee Y., Jin Y.W., Wilkins R.C., Jang S. (2019). Validation of the dicentric chromosome assay for radiation biological dosimetry in South Korea. J. Radiat. Res..

[3] Vral A., Fenech M., Thierens H. (2011). The micronucleus assay as a biological dosimeter of in vivo ionising radiation exposure. Mutagenesis.

[4] Sullivan J.M., Prasanna P.G., Grace M.B., Wathen L.K., Wallace R.L., Koerner J.F., Coleman C.N. (2013). Assessment of biodosimetry methods for a mass-casualty radiological incident: Medical response and management considerations. Health Phys..

[5] Condrat C.E., Thompson D.C., Barbu M.G., Bugnar O.L., Boboc A., Cretoiu D., Suciu N., Cretoiu S.M., Voinea S.C. (2020). miRNAs as Biomarkers in Disease: Latest Findings Regarding Their Role in Diagnosis and Prognosis. Cells.

[6] Li J.R., Tong C.Y., Sung T.J., Kang T.Y., Zhou X.J., Liu C.C. (2019). CMEP: A database for circulating microRNA expression profiling. Bioinformatics.

[7] Enelund L., Nielsen L.N., Cirera S. (2017). Evaluation of microRNA Stability in Plasma and Serum from Healthy Dogs. Microrna.

[8] Glinge C., Clauss S., Boddum K., Jabbari R., Jabbari J., Risgaard B., Tomsits P., Hildebrand B., Kääb S., Wakili R. (2017). Stability of Circulating Blood-Based MicroRNAs—Pre-Analytic Methodological Considerations. PLoS ONE.

[9] Friedman R.C., Farh K.K., Burge C.B., Bartel D.P. (2009). Most mammalian mRNAs are conserved targets of microRNAs. Genome Res..

[10] Michlewski G., Cáceres J.F. (2019). Post-transcriptional control of miRNA biogenesis. RNA.

[11] Khvorova A., Reynolds A., Jayasena S.D. (2003). Functional siRNAs and miRNAs exhibit strand bias. Cell.

[12] Hu H.Y., Yan Z., Xu Y., Hu H., Menzel C., Zhou Y.H., Chen W., Khaitovich P. (2009). Sequence features associated with microRNA strand selection in humans and flies. BMC Genom..

[13] Tharmalingam S., Sreetharan S., Brooks A.L., Boreham D.R. (2019). Re-evaluation of the linear no-threshold (LNT) model using new paradigms and modern molecular studies. Chem. Biol. Interact..

[14] Tharmalingam S., Sreetharan S., Kulesza A.V., Boreham D.R., Tai T.C. (2017). Low-Dose Ionizing Radiation Exposure, Oxidative Stress and Epigenetic Programing of Health and Disease. Radiat. Res..

[15] Puukila S., Tharmalingam S., Al-Khayyat W., Peterson J., Hooker A.M., Muise S., Boreham D.R., Dixon D.L. (2021). Transcriptomic Response in the Spleen after Whole-Body Low-Dose X-ray Irradiation. Radiat. Res..

[16] Mao A., Zhao Q., Zhou X., Sun C., Si J., Zhou R., Gan L., Zhang H. (2016). MicroRNA-449a enhances radiosensitivity by downregulation of c-Myc in prostate cancer cells. Sci. Rep..

[17] Duan X.M., Liu X.N., Li Y.X., Cao Y.Q., Silayiding A., Zhang R.K., Wang J.P. (2019). MicroRNA-498 promotes proliferation, migration, and invasion of prostate cancer cells and decreases radiation sensitivity by targeting PTEN. Kaohsiung J. Med. Sci..

[18] Körner C., Keklikoglou I., Bender C., Wörner A., Münstermann E., Wiemann S. (2013). MicroRNA-31 sensitizes human breast cells to apoptosis by direct targeting of protein kinase C epsilon (PKCepsilon). J. Biol. Chem..

[19] Zaleska K., Przybyła A., Kulcenty K., Wichtowski M., Mackiewicz A., Suchorska W., Murawa D. (2017). Wound fluids affect miR-21, miR-155 and miR-221 expression in breast cancer cell lines, and this effect is partially abrogated by intraoperative radiation therapy treatment. Oncol. Lett..

[20] Chaudhry M.A., Sachdeva H., Omaruddin R.A. (2010). Radiation-induced micro-RNA modulation in glioblastoma cells differing in DNA-repair pathways. DNA Cell Biol..

[21] Maia D., de Carvalho A.C., Horst M.A., Carvalho A.L., Scapulatempo-Neto C., Vettore A.L. (2015). Expression of miR-296-5p as predictive marker for radiotherapy resistance in early-stage laryngeal carcinoma. J. Transl. Med..

[22] Stanbridge E.J., Flandermeyer R.R., Daniels D.W., Nelson-Rees W.A. (1981). Specific chromosome loss associated with the expression of tumorigenicity in human cell hybrids. Somatic. Cell Genet..

[23] Stanbridge E.J., Wilkinson J. (1980). Dissociation of anchorage independence form tumorigenicity in human cell hybrids. Int. J. Cancer.

[24] Pirkkanen J., Tharmalingam S., Morais I.H., Lam-Sidun D., Thome C., Zarnke A.M., Benjamin L.V., Losch A.C., Borgmann A.J., Sinex H.C. (2019). Transcriptomic profiling of gamma ray induced mutants from the CGL1 human hybrid cell system reveals novel insights into the mechanisms of radiation-induced carcinogenesis. Free Radic. Biol. Med..

[25] Pirkkanen J.S., Boreham D.R., Mendonca M.S. (2017). The CGL1 (HeLa × Normal Skin Fibroblast) Human Hybrid Cell Line: History of Ionizing Radiation Induced Effects on Neoplastic Transformation and Novel Future Directions in SNOLAB. Radiat. Res..

[26] Chaudhry M.A., Omaruddin R.A., Brumbaugh C.D., Tariq M.A., Pourmand N. (2013). Identification of radiation-induced microRNA transcriptome by next-generation massively parallel sequencing. J. Radiat. Res..

[27] Campbell J.D., Liu G., Luo L., Xiao J., Gerrein J., Juan-Guardela B., Tedrow J., Alekseyev Y.O., Yang I.V., Correll M. (2015). Assessment of microRNA differential expression and detection in multiplexed small RNA sequencing data. RNA.

[28] Abnizova1 I., Boekhorst R., Orlov Y.L. (2017). Computational Errors and Biases in Short Read Next Generation Sequencing. J. Proteomics Bioinform..

[29] Hulley E.N., Tharmalingam S., Zarnke A., Boreham D.R. (2019). Development and validation of probe-based multiplex real-time PCR assays for the rapid and accurate detection of freshwater fish species. PLoS ONE.

[30] Livak K.J., Schmittgen T.D. (2001). Analysis of relative gene expression data using real-time quantitative PCR and the 2(-Delta Delta C(T)) Method. Methods.

[31] Love M.I., Huber W., Anders S. (2014). Moderated estimation of fold change and dispersion for RNA-seq data with DESeq2. Genome Biol..

[32] Chen Y., Wang X. (2020). miRDB: An online database for prediction of functional microRNA targets. Nucleic Acids Res..

[33] Wang B.D., Ceniccola K., Yang Q., Andrawis R., Patel V., Ji Y., Rhim J., Olender J., Popratiloff A., Latham P. (2015). Identification and Functional Validation of Reciprocal microRNA-mRNA Pairings in African American Prostate Cancer Disparities. Clin. Cancer Res..

[34] Cifuentes-Bernal A.M., Pham V.V., Xiaomei L., Lin L., Jiuyong L., Thuc D.L. (2021). A pseudotemporal causality approach to identifying miRNA–mRNA interactions during biological processes. Bioinformatics.

[35] Santivasi W.L., Xia F. (2014). Ionizing radiation-induced DNA damage, response, and repair. Antioxid Redox. Signal..

[36] Saville M.K., Sparks A., Xirodimas D.P., Wardrop J., Stevenson L.F., Bourdon J.C., Woods Y.L., Lane D.P. (2004). Regulation of p53 by the ubiquitin-conjugating enzymes UbcH5B/C in vivo. J. Biol. Chem..

[37] Pant V., Lozano G. (2014). Limiting the power of p53 through the ubiquitin proteasome pathway. Genes Dev..

[38] Chen J. (2016). The Cell-Cycle Arrest and Apoptotic Functions of p53 in Tumor Initiation and Progression. Cold Spring Harb. Perspect. Med..

[39] Junttila M.R., Puustinen P., Niemelä M., Ahola R., Arnold H., Böttzauw T., Ala-aho R., Nielsen C., Ivaska J., Taya Y. (2007). CIP2A inhibits PP2A in human malignancies. Cell.

[40] Cho U.S., Xu W. (2007). Crystal structure of a protein phosphatase 2A heterotrimeric holoenzyme. Nature.

[41] Mochida S., Ikeo S., Gannon J., Hunt T. (2009). Regulated activity of PP2A-B55 delta is crucial for controlling entry into and exit from mitosis in Xenopus egg extracts. EMBO J..

[42] Yu S., Li L., Wu Q., Dou N., Li Y., Gao Y. (2018). PPP2R2D, a regulatory subunit of protein phosphatase 2A, promotes gastric cancer growth and metastasis via mechanistic target of rapamycin activation. Int. J. Oncol..

[43] Adams D.G., Coffee R.L., Zhang H., Pelech S., Strack S., Wadzinski B.E. (2005). Positive regulation of Raf1-MEK1/2-ERK1/2 signaling by protein serine/threonine phosphatase 2A holoenzymes. J. Biol. Chem..

[44] Baghdoyan S., Lamartine J., Castel D., Pitaval A., Roupioz Y., Franco N., Duarte M., Martin M.T., Gidrol X. (2005). Id2 reverses cell cycle arrest induced by {gamma}-irradiation in human HaCaT keratinocytes. J. Biol. Chem..

[45] Fukuma M., Okita H., Hata J.I., Umezawa A. (2003). Upregulation of Id2, an oncogenic helix-loop-helix protein, is mediated by the chimeric EWS/ets protein in Ewing sarcoma. Oncogene.

[46] Ruiz-Losada M., González R., Peropadre A., Gil-Gálvez A., Tena J.J., Baonza A., Estella C. (2022). Coordination between cell proliferation and apoptosis after DNA damage in *Drosophila*. Cell Death Differ..

[47] Valadi H., Ekström K., Bossios A., Sjöstrand M., Lee J.J., Lötvall J.O. (2007). Exosome-mediated transfer of mRNAs and microRNAs is a novel mechanism of genetic exchange between cells. Nat. Cell Biol.

